# Early postoperative radiotherapy is associated with improved outcomes over late postoperative radiotherapy in the management of completely resected (R0) Stage IIIA-N2 non-small cell lung cancer

**DOI:** 10.18632/oncotarget.18071

**Published:** 2017-05-23

**Authors:** Huan-Huan Wang, Lei Deng, Qing-Lian Wen, Chun-Ze Zhang, Nicholas G. Zaorsky, Bai-Lin Zhang, Jie Chen, Xian-Liang Zeng, Yao-Li Cui, Yang-Yang Shi, Hai-Ling Hou, Wei Wang, Bo Jiang, Jun Wang, Qing-Song Pang, Lu-Jun Zhao, Zhi-Yong Yuan, Ping Wang, Mao-Bin Meng

**Affiliations:** ^1^ Department of Radiation Oncology and CyberKnife Center, Tianjin's Clinical Research Center for Cancer and Key Laboratory of Cancer Prevention and Therapy, Tianjin Medical University Cancer Institute & Hospital, National Clinical Research Center for Cancer, Tianjin 300060, China; ^2^ Department of Thoracic Cancer, Cancer Center and State Key Laboratory of Biotherapy, West China Hospital, West China School of Medicine, Sichuan University, Chengdu 610041, China; ^3^ Department of Oncology, Affiliated Hospital of Southwest Medical University, Luzhou 646000, China; ^4^ Department of Surgery, Nankai University Tianjin People's Hospital, Tianjin People's Hospital, Tianjin 300121, China; ^5^ Department of Radiation Oncology, Fox Chase Cancer Center, Philadelphia, PA 19111, USA; ^6^ Department of Lymphoma, Tianjin's Clinical Research Center for Cancer and Key Laboratory of Cancer Prevention and Therapy, Tianjin Medical University Cancer Institute & Hospital, National Clinical Research Center for Cancer, Tianjin 300060, China; ^7^ Stanford University School of Medicine, Stanford, CA 94305, USA

**Keywords:** non-small cell lung cancer, postoperative radiotherapy, postoperative chemotherapy, multimodality therapy, overall survival

## Abstract

**Aims:**

The aim of this study was to evaluate the ideal timing of PORT in the management of completely resected (R0) Stage IIIA-N2 NSCLC.

**Patients and Methods:**

Between January 2008 and December 2015, patients with known histologies of pathologic Stage IIIA-N2 NSCLC who underwent R0 resection and received PORT concurrent with or prior to two sequential cycles of chemotherapy (“early PORT”) or with PORT administered after two cycles of chemotherapy (“late PORT”) at multiple hospitals. The primary endpoint was OS; secondary end points included pattern of the first failure, LRRFS, and DMFS. Kaplan–Meier OS, LRRFS, and DMFS curves were compared with the log-rank test. Cox regression analysis was used to determine prognosticators for OS, LRRFS, and DMFS.

**Results:**

Of 112 included patients, 41 (36.6%) and 71 (63.4%) patients received early PORT and late PORT, respectively. The median OS, LRRFS, and DMFS were longer for those who received early PORT than for those who received late PORT at the median follow-up of 29.6 months (all *p* < 0.05). Uni- and multi-variate analyses showed that number of POCT cycles and the combination schedule of PORT and POCT were independent prognostic factors for OS, LRRFS, and DMFS.

**Conclusions:**

Early PORT is associated with improved outcomes in pathologic Stage IIIA-N2 R0 NSCLC patients.

## INTRODUCTION

Surgery is a treatment option for certain NSCLC patients, including those with localized (i.e. Stage I-II) and few patients locally advanced (i.e. Stage IIIA) disease. Currently, the NCCN recommends post-surgical observation only in pT1ab R0 or pT2a R0 patients. In contrast, the vast majority of post-operative NSCLC patients are recommended to receive POCT, with or without PORT. POCT is indicated in patients with T2a+ and N1+ disease. PORT is indicated if there is presence of pN2 disease, a positive margin (R+; e.g. R1 or R2), or ECE [1].

In patients with R0 disease who have indications for POCT and PORT, POCT is typically delivered prior to PORT (termed “late PORT” in this manuscript) because these patients are thought to likely harbor micrometastatic disease with a relatively low risk of locoregional disease that would cause a LRR. moreover, such patients would still receive PORT after POCT to prevent LRR. In contrast, the only subsets of patients where PORT is delivered concurrently with POCT or prior to POCT (termed “early PORT”). In these patients, the burden of local disease is theorized to outweigh the risk of micrometastatic disease; PORT is theorized to minimize further micrometastatic dissemination and prevent LRR. Nonetheless, the exact timing of PORT in relationship to POCT has not been investigated in R0 patients.

The aim of this study was to evaluate the ideal timing of PORT in the management of completely resected (R0) Stage IIIA-N2 NSCLC. We hypothesized that the delivery of early PORT in R0 patients would improve patient outcomes.

## RESULTS

### Patient characteristics

**Figure 1:**
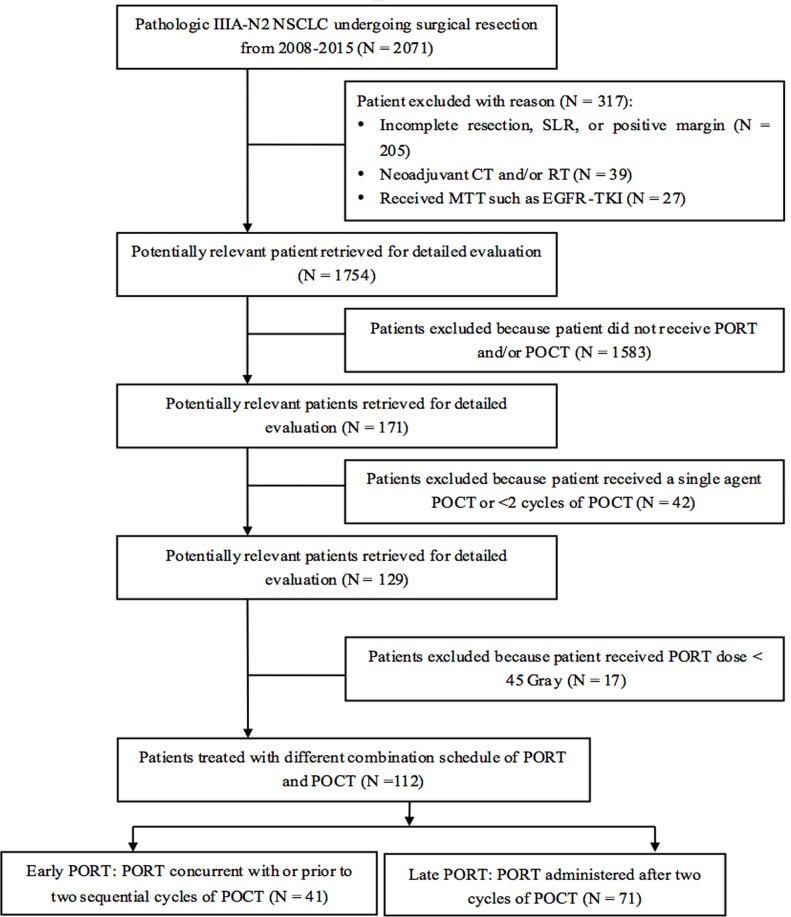
Patient selection NSCLC: non-small cell lung cancer; SLR: sublobar lung resection; CT: chemotherapy; RT: radiotherapy; MTT: molecular targeted therapies; EGFR-TKI: epidermal growth factor tyrosine kinase inhibitors; MLN: mediastinal lymph node; PORT: postoperative radiotherapy; POCT: postoperative chemotherapy.

A total of 112 patients treated between January 1, 2008 and December 30, 2015 were included by a multidisciplinary tumor board from multiple hospitals. Of all eligible patients whose records were examined, 41 (36.6%) and 71 (63.4%) patients were assigned to the early PORT and late PORT, respectively. There were no significant differences in gender, age, smoking history, COPD history, ECOG PS score, tumor location, tumor histology, type of surgery, T classification, number of positive N2 MLNs, positive N2 MLN ratio, number of N2 MLN stations, the interval between surgery and POCT, and total dose of PORT between the two groups (all *p* > 0.05). Differences were observed for the interval between surgery and PORT, the number of POCT cycles and POCT regimens (*p* <0.05). The selection of patients and the baseline characteristics of all patients are shown in Figure 1 and Table 1.

**Table 1:** Patient characteristics

Characteristics	N (%)			χ^2^ values	*p* values
	Overall (N = 112)	Early PORT (N = 41)	Late PORT (N =71)		
Gender					
Male	77 (68.8)	32 (78.1)	45 (63.4)	2.60	0.11
Female	35 (31.2)	9 (21.9)	26 (36.6)		
Age (median years)					
≤ 58	59 (52.7)	19 (46.3)	40 (56.3)	1.04	0.31
> 58	53 (47.3)	22 (53.7)	31 (43.7)		
Smoking history					
Yes	69 (61.6)	29 (70.7)	40 (56.3)	2.28	0.13
No	43 (38.4)	12 (29.3)	31 (43.7)		
COPD history^†^					
Yes	4 (3.6)	2 (4.9)	2 (2.8)	0.32	0.57
No	108 (96.4)	39 (95.1)	69 (97.2)		
ECOG-PS score^†^					
0-1	111 (99.1)	40 (97.6)	71 (100)	2.03	0.16
2	1 (0.9)	1 (2.4)	0		
Tumor location					
RUL	30 (26.8)	12 (29.3)	18 (25.4)	2.23	0.69
RML	5 (4.5)	2 (4.9)	3 (4.2)		
RLL	24 (21.4)	7 (17.1)	17 (23.9)		
LUL	32 (28.5)	10 (24.4)	22 (31.0)		
LLL	21 (18.8)	10 (24.3)	11 (15.5)		
Tumor histology					
Squamous cell	42 (37.5)	18 (43.9)	24 (33.8)	4.58	0.10
Adenocarcinoma	55 (49.1)	15 (36.6)	40 (56.3)		
Others	15 (13.4)	8 (19.5)	7 (9.9)		
Type of surgery^†^					
Lobectomy	111 (99.1)	41 (100)	70 (98.6)	0.92	0.34
Ipsilateral pneumonectomy	1 (0.9)	0	1 (1.4)		
T classification					
T1	32 (28.6)	14 (34.2)	18 (25.4)	2.08	0.56
T2	55 (49.1)	17 (41.5)	38 (53.5)		
T3	17 (15.2)	6 (14.6)	11 (15.5)		
T4	8 (7.1)	4 (9.7)	4 (5.6)		
Number of dissected N2 nodes					
< 9	41 (36.6)	15 (36.6)	26 (36.6)	0.00001	1.00
≥ 9	71 (63.4)	26 (63.4)	45 (63.4)		
Number of positive N2 MLNs					
Single	45 (40.2)	15 (36.6)	30 (42.3)	0.35	0.56
Multiple	67 (59.8)	26 (63.4)	41 (57.7)		
Positive N2 MLN ratio					
< 25%	54 (48.2)	23 (56.1)	31 (43.7)	1.61	0.21
≥ 25%	58 (51.8)	18 (43.9)	40 (56.3)		
Number of N2 MLN positive stations					
Single	59 (52.7)	21 (51.2)	38 (53.5)	0.06	0.81
Multiple	53 (47.3)	20 (48.8)	33 (46.45)		
Interval between surgery and POCT (months)	1.3 ± 0.6	1.4 ± 0.8	1.2 ± 0.5	1.14	0.26
Interval between surgery and PORT (months)	4.9 ± 2.6	3.2 ± 1.1	5.9 ± 2.8	4.99	**0.0001**
Total dose of PORT					
< 50 Gy	5 (4.4)	0	5 (7.0)	3.02	0.08
≥ 50 Gy	107 (95.6)	41 (100)	66 (93.0)		
POCT regimen^‡^					
TP/DP	53 (47.3)	26 (63.4)	27 (38.0)	14.16	**0.003**
CP	40 (25.7)	14 (34.2)	26 (36.6)		
GP/GC	18 (16.1)	1 (2.4)	17 (23.9)		
NP	1 (0.9)	0	1 (1.5)		
# of POCT cycles					
< 4	15 (13.4)	15 (36.6)	0	29.99	**0.0001**
≥ 4	97 (86.6)	26 (63.4)	71 (100)		
Gene expression status					
EGFR mutation	9 (8.0)	2 (4.9)	7 (9.9)	1.49	0.47
ALK positive	1 (0.9)	0	1 (1.4)		
N/A	102 (91.1)	39 (95.1)	63 (88.7)		
Molecular targeted therapy					
Erlotinib	2 (1.8)	1 (2.4)	1 (1.4)	2.35	0.50
Gefitinib	7 (6.3)	1 (2.4)	6 (8.5)		
Crizotinib	1 (0.9)	0	1 (1.4)		
None	102 (91.0)	39 (95.2)	63 (88.7)		
Treatment strategies after progression					
RT	12 (10.7)	3 (7.3)	9 (12.7)	2.91	0.41
S	20 (17.9)	1 (2.4)	0		
CT	9 (8.0)	9 (22.0)	19 (26.8)		
BSC	71 (63.4)	28 (68.3)	43 (60.5)		

Note: Bold-face denotes *p*-value < 0.05.

^†^Subgroups have less than five patients.

^‡^ Platinum-based CT regimens included cisplatin, carboplatin, or oxaliplatin.

COPD: chronic obstructive pulmonary disease; ECOG-PS: the Eastern Cooperative Oncology Group scale-performance status; RUL: right upper lobe; RML: right middle lobe; RLL: right lower lobe; LUL: left upper lobe; LLL: left lower lobe; TP/DP: paclitaxel or docetaxel + platinum included cisplatin, carboplatin, or oxaliplatin; CP: pemetrexed + platinum included cisplatin, carboplatin, or oxaliplatin; GP/GC: gemcitabine + platinum included cisplatin, carboplatin, or oxaliplatin; NP: vinorelbine + platinum included cisplatin, carboplatin, or oxaliplatin; PORT: postoperative radiotherapy; POCT: postoperative chemotherapy; EGFR: epidermal growth factor receptor; ALK: anaplastic lymphoma kinase; N/A: not report; RT: radiotherapy; CT: chemotherapy; S: surgery; BSC: best supportive care.

### OS, LRRFS, and DMFS

**Figure 2:**
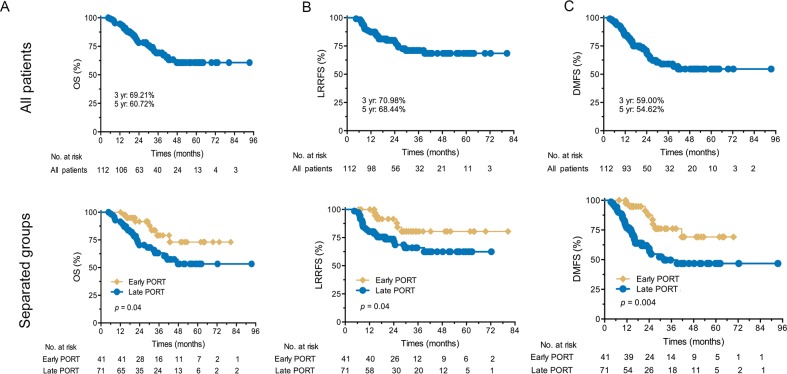
Kaplan–Meier survival curves **(A)** OS for all patients and separate groups; **(B)** LRRFS for all patients and separate groups; **(C)** DMFS for all patients and separate groups. OS: overall survival; yr: year; LRRFS: locoregional recurrence-free survival; DMFS: distant metastasis-free survival; PORT: postoperative radiotherapy; POCT: postoperative chemotherapy.

For the whole cohort, the median OS, LRRFS, and DMFS were not reached during the median follow-up was 29.6 months (range, 4.7–93.5 months) (Figure 2). The 3- and 5-year OS rates were 79.1% and 73.1% in the early PORT, respectively; these were statistically significantly higher than those of late PORT group, with the respective rates of 63.2% and 53.4%. The 3- and 5-year LRFFS rates were 80.5% and 80.5% in the early PORT and 65.9% and 62.4% in the late PORT, respectively, and the differences in LRFFS rates between the two groups trended toward significance. The 3- and 5-year DMFS rates were 76.1% and 69.2% in the early PORT as well as 49.0% and 46.1% in late PORT, respectively, and the differences in DMFS rates between the two groups also were significant (all p < 0.05).

### Patterns of failure and the first failure

Up to the last follow-up, treatment failure was observed in 50 (44.6%) patients, LR occurred in 24 (21.4%) patients, DM occurred in 44 (39.3%) patients, and both LR and DM occurred in 18 (16.1%) patients. LR occurred as the first failure site in 6 (14.6%) and 15 (21.1%) patients in the early and late PORT groups, respectively (*p* = 0.40). DM occurred as the first failure site in 13 (31.7%) patients in the early PORT versus 26 (36.6%) patients in the late PORT (*p* = 0.21). Both LR and DM occurred as the first failure site in 3 (7.3%) and 7 (9.9%) patients in the early and late PORT groups, respectively (*p* = 0.65). In addition, of 44 (39.3%) patients who had DM, the most frequent sites were lung, bone, lung pleura, central nervous system, adrenal gland, liver, and others sequentially. The cumulative incidences of LR and DM are listed in Table 2.

**Table 2:** Pattern of failure and the first failure

Parameters	N (%)			χ^2^ values	*p* values
	All (N = 112)	Early PORT (N = 41)	Late PORT (N = 71)		
LR as first site of failure					
Yes	21 (18.8)	6 (14.6)	15 (21.1)	0.72	0.40
No	91 (81.2)	35 (85.4)	56 (78.9)		
All LR failure					
Yes	24 (21.4)	8 (39.0)	16 (22.5)	0.14	0.71
No	88 (78.6)	33 (61.0)	55 (77.5)		
DM as first site of failure					
Yes	39 (34.8)	13 (31.7)	26 (36.6)	1.56	0.21
No	73 (65.2)	28 (68.3)	45 (63.4)		
All DM failure					
Yes	44 (39.3)	13 (31.7)	31 (43.7)	1.56	0.21
No	68 (60.7)	28 (68.3)	40 (56.3)		
Both LR and DM as first site of failure					
Yes	10 (8.9)	3 (7.3)	7 (9.9)	0.21	0.65
No	102 (91.1)	38 (92.7)	64 (90.1)		
Both LR and DM failure					
Yes	18 (16.1)	5 (12.2)	13 (18.3)	0.72	0.39
No	94 (83.9)	36 (87.8)	58 (81.7)		
Site of DM^†^					
Lung	20 (17.9)	7 (17.1)	13 (18.3)	-	-
Bone	14 (12.5)	3 (7.3)	11 (15.5)		
Lung pleura	7 (6.3)	3 (7.3)	4 (5.6)		
Central nervous system	6 (5.4)	2 (4.9)	4 (5.6)		
Adrenal gland	4 (3.5)	1 (2.4)	3 (4.2)		
Liver	4 (3.5)	2 (4.9)	2 (2.8)		
Others	6 (5.4)	1 (2.4)	5 (7.0)		

Note: some patients had more than one site of LR or/and DM failure at the same time.

PORT: postoperative radiotherapy; LR: locoregional recurrence; DM: distant metastasis.

^†^Fifteen patients concurrent with multiple metastases including lung, bone, lung pleura, central nervous system, adrenal gland, liver, or others.

### Prognostic factors associated with OS, LRRFS, and DMFS

The results of univariate analyses for clinical factors affecting OS are presented in Table 3. The patients with ≥ 4 CT cycles of POCT and those who received early PORT experienced significantly greater OS, LRRFS, and DMFS (all *p* < 0.05). In addition, age (*p* = 0.01), smoking history (*p* = 0.02), the number of POCT cycles (*p* = 0.007), and the combination schedule of PORT and POCT (*p* = 0.04) were significant factors affecting OS; simultaneously, tumor histology (*p* = 0.02 and *p* = 0.03), the number of POCT cycles (*p* = 0.02 and *p* = 0.03), and the combination schedule of PORT and POCT (*p* = 0.04 and *p* = 0.004) were statistically significant clinical factors affecting LRRFS and DMFS, respectively.

**Table 3:** Univariate analyses for clinical variables affecting OS, LRRFS, and DMFS

Variable	OS				LRRFS				DMFS			
	3-yr	5-yr	χ^2^	*p*	3-yr	5-yr	χ^2^	*p*	3-yr	5-yr	χ^2^	*p*
Patient inclusion site												
A	70.4%	65.0%	1.31	0.73	74.0%	74.0%	3.25	0.35	65.7%	60.3%	4.30	0.23
B	64.1%	46.1%			59.8%	59.8%			44.7%	44.7%		
C	63.5%	63.5%			61.5%	61.5%			53.6%	53.6%		
D	76.2%	76.2%			83.1%	83.1%			60.6%	60.6%		
Gender												
Male	63.8%	57.0%	2.02	0.16	73.1%	68.8%	0.01	0.92	54.8%	51.4%	0.66	0.42
Female	71.9%	67.1%			65.8%	65.8%			66.3%	59.7%		
Age												
≤ 58 years	81.1%	70.7%	6.27	**0.01**	68.0%	68.0%	0.04	0.84	57.5%	53.7%	0.02	0.88
> 58 years	52.8%	47.5%			76.1%	69.2%			60.5%	55.0%		
Smoking history												
Yes	58.1%	54.5%	5.27	**0.02**	71.0%	71.0%	0.14	0.71	53.4%	53.4%	0.61	0.44
No	84.1%	69.1%			71.7%	66.9%			65.7%	56.8%		
COPD history^†^												
Yes	75.0%	0	1.84	0.18	100%	100%	1.04	0.31	50.0%	50.0%	0.14	0.71
No	70.2%	61.6%			70.1%	67.5%			58.7%	54.3%		
ECOG-PS score^†^												
0-1	68.5%	60.1%	0.17	0.68	71.8%	69.2%	3.39	0.07	59.0%	54.6%	3.25	0.07
2	100%	100%			0	0			0	0		
Tumor location												
RUL	74.0%	64.1%	9.31	0.05	53.6%	53.6%	2.50	0.65	57.2%	49.0%	4.79	0.31
RML	100%	100%			80.0%	80.0%			80.0%	80.0%		
RLL	60.0%	25.0%			59.1%	59.1%			43.2%	43.2%		
LUL	75.0%	75.0%			72.5%	72.5%			51.8%	51.8%		
LLL	77.9%	77.9%			85.7%	85.7%			82.7%	68.9%		
Tumor histology												
Sqa	78.0%	78.0%	3.28	0.19	88.2%	88.2%	8.43	**0.02**	75.5%	75.5%	7.13	**0.03**
Ade	61.6%	55.4%			59.5%	59.5%			46.3%	46.3%		
Others	81.8%	56.1%			72.9%	72.9%			66.3%	53.0%		
Type of surgery^†^												
Lobectomy	68.2%	59.6%	0.51	4.73	70.6%	68.0%	0.38	0.54	57.9%	53.5%	0.62	0.43
Ipsilateral pneumonectomy	100%	100%			100%	100%			100%	100%		
T classification												
T1	82.9%	76.0%	2.96	0.40	74.4%	74.4%	0.60	0.90	62.9%	62.9%	1.78	0.62
T2	62.1%	55.2%			67.9%	67.9%			52.2%	48.2%		
T3	61.9%	46.5%			81.1%	54.1%			69.3%	52.0%		
T4	85.7%	0			70.0%	70.0%			87.5%	87.5%		
Number of N2 MLN positive nodes												
Single	73.2%	55.8%	0.02	0.90	74.5%	74.5%	0.11	0.74	61.8%	56.6%	0.04	0.84
Multiple	64.6%	64.6%			68.6%	63.7%			55.6%	51.6%		
Positive N2 MLN ratio												
≤ 25%	67.6%	58.7%	0.001	0.97	74.4%	69.1%	0.68	0.41	61.1%	56.4%	1.69	0.19
> 25%	69.5%	61.5%			67.4%	67.4%			55.8%	51.5%		
Number of N2 MLN positive stations												
Single	79.2%	79.2%	3.71	**0.04**	74.1%	74.1%	0.004	0.95	60.1%	55.8%	0.25	0.62
Multiple	61.2%	48.0%			70.4%	65.0%			57.2%	52.5%		
Total dose of PORT^†^												
< 50 Gy	80.0%	80.0%	0.01	0.91	80.0%	80.0%	0.02	0.90	66.7%	66.7%	0.14	0.71
≥ 50 Gy	68.3%	59.7%			70.6%	67.9%			58.3%	53.8%		
Number of POCT cycles												
< 4	45.6%	45.6%	7.41	**0.007**	56.8%	56.8%	5.94	**0.02**	49.1%	49.1%	4.38	**0.03**
≥ 4	73.0%	63.1%			74.0%	71.1%			60.2%	57.2%		
The combination schedule												
Early PORT	79.1%	73.1%	4.19	**0.04**	80.5%	80.5%	4.12	**0.04**	76.1%	69.2%	8.16	**0.004**
Late PORT	63.2%	53.4%			65.9%	62.4%			49.0%	46.1%		

Note: Bold-face denotes *p*-value < 0.05.

^†^ Subgroups have less than five patients.

OS: overall survival; LRRFS: locoregional recurrence-free survival; DMFS: distant metastasis-free survival; MST: median survival time; mo: months; yr: year; COPD: chronic obstructive pulmonary disease; ECOG-PS: the Eastern Cooperative Oncology Group scale-performance status; RUL: right upper lobe; RML: right middle lobe; RLL: right lower lobe; LUL: left upper lobe; LLL: left lower lobe; Sqa: squamous cell; Ade: adenocarcinoma; MLN: mediastinal lymph node; PORT: postoperative radiotherapy; POCT: postoperative chemotherapy.

On multivariate analyses, the statistically significant prognostic factors for OS included tumor histology (HR = 2.186, *p* = 0.04), number of POCT cycles (HR = 0.235, *p* = 0.005), and the combination schedule of PORT and POCT (HR = 0.183, *p* = 0.001). Significant prognostic factors for LRRFS and DMFS included ECOG-PS score (HR = 72.343, *p* = 0.02; HR = 36.565, *p* = 0.004), tumor histology (HR = 2.176, *p* = 0.054; HR = 2.011, *p* = 0.04), number of POCT cycles (HR = 0.131, *p* = 0.0001; HR = 0.292, *p* = 0.009), and the combination schedule of PORT and POCT (HR = 0.196, *p* = 0.005; HR = 0.167, *p* = 0.0001), respectively (Table 4).

**Table 4:** Multivariable analyses for clinical variables affecting OS, LRRFS, and DMFS

Variable	OS			LRRFS			DMFS		
	HR	95% CI	*p* value	HR	95% CI	*p* value	HR	95% CI	*p* value
Patient inclusion site (A vs. B vs. C vs. D)	0.944	0.642-1.387	0.944	0.901	0.570-1.423	0.654	1.193	0.823-1.729	0.351
Gender (female vs. male)	1.155	0.361-3.690	0.808	1.288	0.409-4.057	0.665	0.760	0.303-1.907	0.559
Age (≤ 58 vs. > 58)	1.771	0.795-3.946	0.162	0.701	0.289-1.700	0.432	0.901	0.453-1.793	0.901
Smoking history (yes vs. no)	0.289	0.091-0.920	0.036	0.733	0.232-2.318	0.597	0.735	0.298-1.813	0.503
COPD history (yes vs. no)^†^	0.322	0.059-1.762	0.191	302.604	0.0001-	0.986	1.181	0.148-9.397	0.875
ECOG-PS score (0-1 vs. 2)^†^	0	0	0.992	72.343	4.712-1110.747	**0.002**	36.565	3.151-424.359	**0.004**
Tumor location (RUL vs. RML vs. RLL vs. LUL vs. LLL)	0.954	0.725-1.254	0.734	0.944	0.706-1.262	0.697	1.065	0.835-1.358	0.612
Tumor histology (Sqa vs. Ade vs. others)	2.186	1.036-4.616	**0.040**	2.176	0.988-4.793	0.054	2.011	1.031-3.922	**0.040**
Type of surgery (lobectomy vs. Ipsilateral pneumonectomy)^†^	0	0	0.981	0.0001	0.0001-	0.993	0.0001	0.0001-	0.977
T classification (T1 vs. T2 vs. T3 vs. T4)	1.393	0.871-2.229	0.167	1.125	0.675-1.873	0.652	1.127	0.728-1.744	0.592
Number of N2 MLN positive stations (single vs. multiple)	0.461	0.191-1.114	0.085	0.747	0.309-1.802	0.516	1.041	0.517-2.096	0.911
Total dose of PORT (< 50 Gy vs. ≥ 50 Gy)	8.364	0.748-93.488	0.085	6.951	0.632-76.416	0.113	5.047	0.603-42.208	0.135
Number of POCT cycles (≥ 4vs. < 4)	0.235	0.086-0.640	**0.005**	0.131	0.043-0.400	**0.0001**	0.292	0.117-0.732	**0.009**
The combination schedule (early PORT vs. late PORT)	0.183	0.066-0.511	**0.001**	0.196	0.063-0.611	**0.005**	0.167	0.064-0.434	**0.0001**

^†^ Subgroups have less than five patients.

Note: Bold-face denotes *p*-value < 0.05.

OS = overall survival; LRRFS: locoregional recurrence-free survival; DMFS: distant metastasis-free survival; HR: hazard ratio; CI: confidence interval; COPD: chronic obstructive pulmonary disease; ECOG-PS: the Eastern Cooperative Oncology Group scale-performance status; Sqa: squamous cell; Ade: adenocarcinoma; RUL: right upper lobe; RML: right middle lobe; RLL: right lower lobe; LUL: left upper lobe; LLL: left lower lobe; MLN: mediastinal lymph node; PORT: postoperative radiotherapy; POCT: postoperative chemotherapy.

### Toxicities

Twelve patients (10.7%) experienced CTCAE v4.0 Grade 1 to 2 acute toxicities including pneumonitis, esophagitis, chest pain, agranulocytosis, and throm-bocytopenia. Five patients (4.5%) experienced Grade 3 acute toxicities including esophagitis and tracheitis. Almost all of these acute toxicities occurred in the early PORT group, and they were generally transient and resolved with conservative management. Late radiation toxicities were observed in two patients (1.8%) including pulmonary fibrosis, and both patients were in the early PORT. None of the patients died from Grade 5 late toxicities.

## DISCUSSION

With improvements in radiotherapy equipment and techniques, several clinicians have investigated the efficacy and safety of PORT for patients with resected NSCLC and demonstrated decreased survival in the subset of N0-1 NSCLC patients who received PORT. However, in patient with pathologic N2 disease, the use of PORT was associated with a significant improvement in OS without serious toxicities [2–7]. Although it has yet to be proven in randomized trials, the NCCN recommends a sequence of POCT and PORT for patients with pathologic Stage IIIA-N2 NSCLC, because PORT has been incorporated into multidisciplinary management to improve locoregional control in resected Stage IIIA-N2 NSCLC, which may further translate into a survival benefit. However, the optimal schedule of PORT and POCT remains poorly understood and warrants further investigation for patients with pathologic stage IIIA-N2 NSCLC.

There has been no study comparing the optimal sequencing of POCT and PORT among patients with pathologic Stage IIIA-N2 R0 NSCLC [8]. In the absence of randomized data, we sought to answer this question in a multi-institutional retrospective study of high-quality data to provide insight into the relationship between the PORT and POCT combination schedule and survival. In this study, we found that pathologic Stage IIIA-N2 R0 NSCLC patients treated with early PORT had better OS, LRRFS, and DMFS than those treated with late PORT. Thus, early PORT is associated with improved outcomes in pathologic Stage IIIA-N2 R0 NSCLC. Our study provides new evidence to optimize the postoperative treatment strategy for patients with pathologic Stage IIIA-N2 NSCLC. Prospective studies must be performed to confirm the optimal schedule of PORT and POCT in the treatment of patients with pathologic Stage IIIA-N2 NSCLC.

In the definitive treatment for locally advanced NSCLC, concurrent POCT and PORT has been proven to be superior to RT alone [9], as well as to sequential chemotherapy followed by RT [10]. Some phase II studies have shown that postoperative concurrent POCT and PORT provide promising results both in terms of treatment-related toxicities and survival [11–13]. However, other studies report no improvement in outcomes with concurrent POCT and PORT vs either treatment alone [13–14]. Further, the IAEA stated that PORT represents a form of elective nodal irradiation (ENI), and this concept has been abandoned gradually in clinical practice despite a lack of clear evidence for such an approach [15–16]. These results led us to presume that a suboptimal timing of POCT and PORT cause their inefficacy in these patients.

The ACR guidelines recommend delivering PORT sequentially after completion of POCT because POCT would address micrometastatic disease, and DMs are a prevailing failure pattern among these patients [17]. For example, Dautzenberg *et al*. reported on 267 patients (259 with Stage II and III disease) who were randomized to PORT with 60 Gray or POCT followed by PORT [18]. There was no difference in DFS or OS between the two arms; DMs occurred more frequently in the RT group (*p* = 0.09) whereas LR occurred similarly in both groups (*p* = 0.27). Additionally, in a retrospective study of 105 patients with Stage IIIA-N2 NSCLC, a PORT-first strategy after surgery appeared not to compromise the clinical outcomes. The benefit of POCT on OS, with or without PORT first [19]. Together, these previous findings indicate that the optimal combination schedule of PORT and POCT remains poorly understood and warrants further investigation.

Our multi-institutional retrospective study showed that the median OS was longer for those who received early PORT than for those who received late PORT. The rationale for early PORT comes from multiple factors. First, locoregional tumor burden is assumed to be higher than that of distant micrometastases in patients with pN2 disease [20]. Second, PORT tends to achieve a better tumor response rate when compared to historical trials using POCT [21–23]; thus, delaying PORT may lead to the loss of optimal time for controlling the locoregional residual tumor. Third, the addition of POCT after surgery for patients with operable NSCLC improves OS, irrespective of whether POCT was adjuvant to surgery alone or adjuvant to surgery plus PORT [24].

Recently, Lee *et al*. demonstrated that the PORT followed by POCT might be more effective in terms of locoregional control without compromising OS for Stage IIIA-N2 NSCLC [19]. The 5-year OS of the early PORT in the present study was 60.7%, which is similar to their reported value of 61.3%. In the study by Lee *et al*., one of the most important questions about sequential PORT followed by POCT was whether postponing POCT might be deleterious to survival. In our study, there were 15 patients who received fewer than 4 cycles of POCT (Table 1), and this may compromise its efficacy. Therefore, the combination schedule of PORT and POCT warrant further investigation for patients with pathologic Stage IIIA-N2 NSCLC.

Detailed investigation of the patterns of failure after treatment enables the identification of optimum treatments. In the present study, the combined event rate for LR and DM of almost 44.6% (50 patients), and the plurality of failures were DMs (Table 2). This failure pattern is likely reflective of the relatively large number of patients with adenocarcinoma as the primary histologic subtype (Table 1). There were no differences between the two groups in terms of LR, DM, and both LR and DM as relapse or the first site of relapse (all *p* > 0.05, Table 2); nonetheless, our analysis revealed that early PORT, compared with late PORT, was associated with improved OS, LRRFS, and DMFS (all *p* < 0.05, Table 3). One possible reason might be that early PORT exterminated the existing small tumors and reduced the possibility of these small tumors to spread to remote locations. Furthermore, multivariate analyses showed that ≥ 4 cycles of POCT and early PORT were favorable prognostic factors for OS, LRRFS, and DMFS (all *p* < 0.05, Table 4).

The appropriate dose in the PORT has not been addressed in a randomized trial. The required dose for sites of potential occult disease may vary depending on the probability of residual disease, the number of sites at risk, and the desired control rate. Together, the results of our study along with those of previous studies suggest that PORT doses of 45 Gy or higher are well tolerated when given with two different combination schedules of PORT and POCT. It should be noted that the older randomized trials using this dose found no survival benefit, presumably due to excess toxicity related to PORT. By comparison, modern radiotherapy equipment and techniques including 3D-CRT, IMRT, and VMAT have been widely adapted in several clinical areas in an effort to improve dose homogeneity and target coverage, and to decrease normal tissue exposure in comparison to outdated radiation equipment and techniques.

In addition, although growing evidence suggests that PORT administered using the modern PORT technique has a favorable effect on the survival of patients with N2 disease, there exists significant heterogeneity within the reported studies with respect to the irradiation fields employed for PORT and the consensus guidelines regarding the dose and CTV [25–28]. Recently, Feng *et al*. designed a patterns-of-failure study after R0 surgery in resected N2 disease to evaluate the rationale of the proposed PORT CTVs based on the most likely sites of nodal failure, and the institutional standard CTV delineation for PORT was developed in their hospital [29–30]. Similar to these studies, our CTV encompassed the bronchial stump and involved mediastinal nodal stations and their next draining stations.

The present study does have potential weakness. First, the study carries with it all of the limitations inherent to a retrospective analysis. Second, the sample size of the present study was relatively small; however, patients with pathologic Stage IIIA-N2 R0 NSCLC treated with PORT were rare during the past decades when the PORT meta-analysis was published. Third, this study did analyze RT dose (<50 Gy and ≥50 Gy) as a variable for OS, LRRFS, and DMFS in uni- and multivariate analyses, and the effect of the RT dose did not seem to be significant. However, more specific RT details such as dose to normal tissues need to be reported. Additionally, these are more heterogeneity in the IIIA-N2 patients. Some patients are optional to fail in distant metastases, others patients more in local-regional regions. For example, the late PORT group included more adenocarcinoma patients with more DM. This may explain the more DM in late PORT group. Finally, we acknowledge that the method by which patients were chosen to receive the different combination schedule of PORT and POCT was not random but rather influenced by patients’ and physicians’ preferences, baseline characteristics, and practice patterns; therefore, it may have contributed to study bias.

## PATIENTS AND METHODS

### Study design and eligible patients

We queried a retrospective database of patients with known histologies of pathologic Stage IIIA-N2 NSCLC from multiple hospitals. Patients were treated between January 1, 2008 and December 30, 2015. All patients were examined in a multidisciplinary setting by surgical, medical, and radiation oncologists at the time of diagnosis, and their cases were re-presented in front of the tumor board on an as-needed basis (e.g., postoperatively).

The inclusion criteria were defined as follows: (I) any age; (II) KPS ≥ 70 (assessed pre- and post-operatively); (III) pathologic Stage IIIA-N2 NSCLC with histologic confirmation (assessed post-operatively); (IV) life expectancy >6 months in order to exclude perioperative mortality (assessed pre-operatively); (V) receipt of surgical resection with negative margins (R0) plus complete MLN dissection, PORT, and POCT; and (VI) written informed consent for the treatment and inclusion in the database.

The exclusion criteria were as follows: (I) non pathological Stage IIIA-N2 NSCLC; (II) sublobar lung resection, no radical resection, or incomplete MLN dissection; (III) use of neoadjuvant chemotherapy and/or radiotherapy; (IV) use of molecular targeted therapies such as epidermal growth factor tyrosine kinase inhibitors (EGFR-TKIs), etc.; (V) receipt of single agent of POCT or <2 cycles of POCT; (VI) PORT dose < 45 Gray and/or with palliative RT intent; (VII) evidence of metastatic disease; (VII) contraindication to receiving combination therapy (e.g. change in performance status); and (VIII) uncontrolled comorbid conditions (metabolic or psychiatric). The study protocol was designed in accordance with the ethical guidelines of the Declaration of Helsinki and was approved by the independent ethics committees at all participating hospitals.

### Treatment schedule

**Figure 3:**
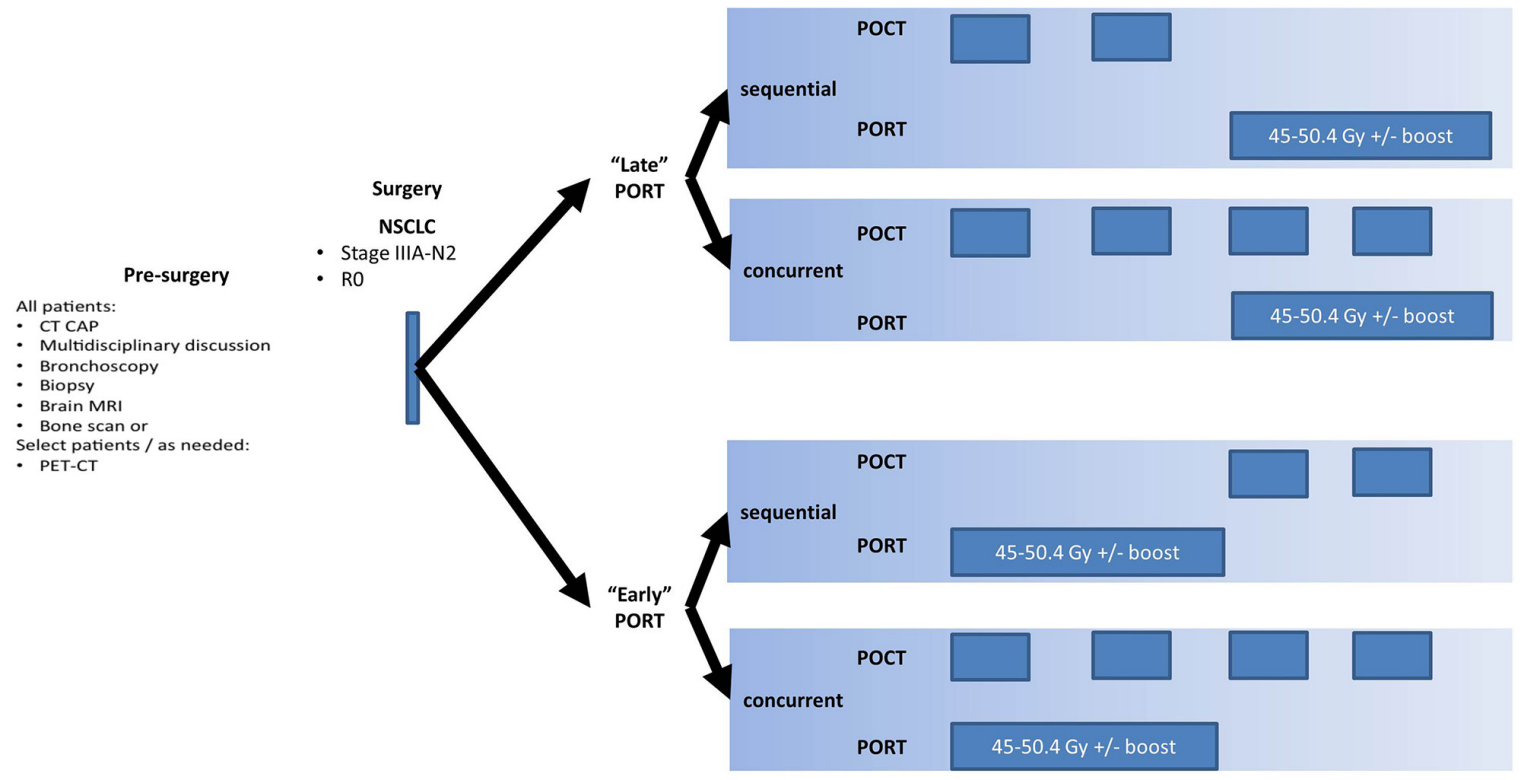
Treatment scheme and definition of “early” and “late” PORT

The treatment schedule and definitions of early vs. late PORT are shown in Figure 3. For baseline staging, bronchoscopy, computed tomography scanning of the chest and upper abdomen, brain MRI with enhancement, and bone scintigraphy or PET-CT were routinely performed. All patients underwent lobectomy or ipsilateral pneumonectomy as well as complete MLN dissection. In addition, the administration of PORT for a patient with pathologic Stage IIIA-N2 disease was based on the attending radiation oncologist's decision and, partially, the referring surgeon's suggestion.

PORT treatment was per our institutional guidelines, which were applicable to all patients fitting inclusion criteria. PORT was administered with 3D-CRT, IMRT, or VMAT. The initial CTV encompassed a bronchial stump, involved the mediastinal nodal stations and the next draining stations. The boost CTV included a bronchial stump and involved nodal stations only. The PTV was extended in all directions from the CTV by a margin of 1–1.5 cm. The tumor bed was included only if invasion of the parietal pleura was documented in the operative report. Neither the contralateral hilum nor the supraclavicular fossae were included on a routine basis. If, however, it was necessary to treat the tumor bed for a lesion of the upper lobe, the supraclavicular fossae were included. Conventional fractionation (1.8–2.0 Gray/day) was used with a dose of 45–50.4 Gray for the initial volume, and the boost volume was irradiated up to 60 Gray according to the risk of recurrence.

Data gathered for POCT included agent(s) used and the number of POCT cycles, and the combination schedule of PORT and POCT was extracted for each patient. Per Figure 2, included patients were divided (1) “early PORT,” which was defined by a regimen in which patients received PORT concurrent with POCT, or following two cycles of POCT; (2) and “late PORT,” which was defined by a regimen in which patients received two cycles of POCT without PORT, and then received PORT (with or without concurrent chemotherapy).

### Follow-up

Patients were seen in clinic at 1 month after completion of treatment, then every 3 months for the first year, and then, every 6 months until April 20, 2016. Imaging, adverse events, and the compliance of all patients were monitored during the follow-up period using our clinical databases.

### Endpoints

The primary aim was OS. The secondary aims were: (i) pattern of the first failure; (ii) locoregional recurrence-free survival (LRRFS); (iii) distant metastasis-free survival (DMFS); and (iv) toxicity according to the CTCAE v4.0. OS was defined as the time between the date of diagnosis and the date of death or the date of the last follow-up for censored patients. The LRRFS and DMFS were defined as the time between the date of the post-operation and the date of the locoregional recurrence and/or distant metastases or the last follow-up for censored patients. To prevent immortal time bias, all patients needed to receive a minimum of 2 cycles of POCT and must have been alive for at least 1 month after surgery [31]. All toxicities were assessed in a multidisciplinary setting. Locoregional failure was defined as tumor regrowth in the hilar, mediastinal, or supraclavicular lymph nodes or at the bronchial margin of resection, as visualized by computed tomography or PET-CT scanning. Recurrences beyond these sites were deemed distant metastases. In addition, PET-CT scanning was employed to assist with differentiating radiation-related changes with recurrence and/or metastasis.

### Statistical analysis

The χ^2^ test or Fisher's exact test was performed for qualitative data. OS, LRRFS, and DMFS curves were estimated by using the Kaplan–Meier technique and compared by the stratified log-rank test. Uni- and multi-variate analyses were performed using a Cox regression model. Data were analyzed using Intercooled Stata, version 8.2 for Windows (Stata Corporation, College station, Texas, USA), with a *p* value of < 0.05 considered significant.

## Abbreviations

PORT: postoperative radiotherapy
NSCLC: non-small cell lung cancer
OS: overall survival
LRRFS: locoregional recurrence-free survival
DMFS: distant metastasis-free survival
POCT: postoperative chemotherapy
NCCN: national comprehensive cancer network
ECE: extracapsular extension
LRR: locoregional recurrence
KPS: karnofsky performance score
MLN: mediastinal lymph node
EGFR-TKIs: epidermal growth factor tyrosine kinase inhibitors
MRI: magnetic resonance imaging
PET: positron emission tomography
3D-CRT: three-dimensional conformal radiotherapy
IMRT: intensity-modulated radiotherapy
VMAT: volumetric modulated arc therapy
CTV: clinical target volume
PTV: planning target volume
HR: hazard ratio
IAEA: international atomic energy agency
ENI: elective nodal irradiation
ACR: The American college of radiology
CTCAE: common terminology criteria for adverse events.
